# Molecular mechanisms of Wischnewski spot development on gastric mucosa in fatal hypothermia: an experimental study in rats

**DOI:** 10.1038/s41598-020-58894-8

**Published:** 2020-02-05

**Authors:** Chihpin Yang, Kana Sugimoto, Yukie Murata, Yuichiro Hirata, Yu Kamakura, Yoshihisa Koyama, Yohei Miyashita, Kentaro Nakama, Kazuma Higashisaka, Kazuo Harada, Ryuichi Katada, Hiroshi Matsumoto

**Affiliations:** 1 Department of Legal Medicine, Osaka University Graduate School of Medicine, Osaka, Japan; 2 Department of Neuroscience and Cell Biology, Osaka University Graduate School of Medicine, Osaka, Japan

**Keywords:** Mechanisms of disease, Experimental models of disease

## Abstract

Numerous dark-brown-coloured small spots called “Wischnewski spots” are often observed in the gastric mucosa in the patients dying of hypothermia, but the molecular mechanisms through which they develop remain unclear. We hypothesised that hypothermia may activate the secretion of gastric acid and pepsin, leading to the development of the spots. To investigate this, we performed experiments using organotypic rat gastric tissue slices cultured at 37 °C (control) or 32 °C (cold). Cold loading for 6 h lowered the extracellular pH in the culture medium. The mRNA expression of gastrin, which regulates gastric acid secretion, increased after cold loading for 3 h. Cold loading increased the expression of gastric H^+^,K^+^-ATPase pump protein in the apical canalicular membrane and resulted in dynamic morphological changes in parietal cells. Cold loading resulted in an increased abundance of pepsin C protein and an elevated mRNA expression of its precursor progastricsin. Collectively, our findings clarified that cold stress induces acidification by activating gastric H^+^,K^+^-ATPase pumps and promoting pepsin C release through inducing progastricsin expression on the gastric mucosa, leading to tiny haemorrhages or erosions of the gastric mucosa that manifest as Wischnewski spots in fatal hypothermia.

## Introduction

**Figure 1:**
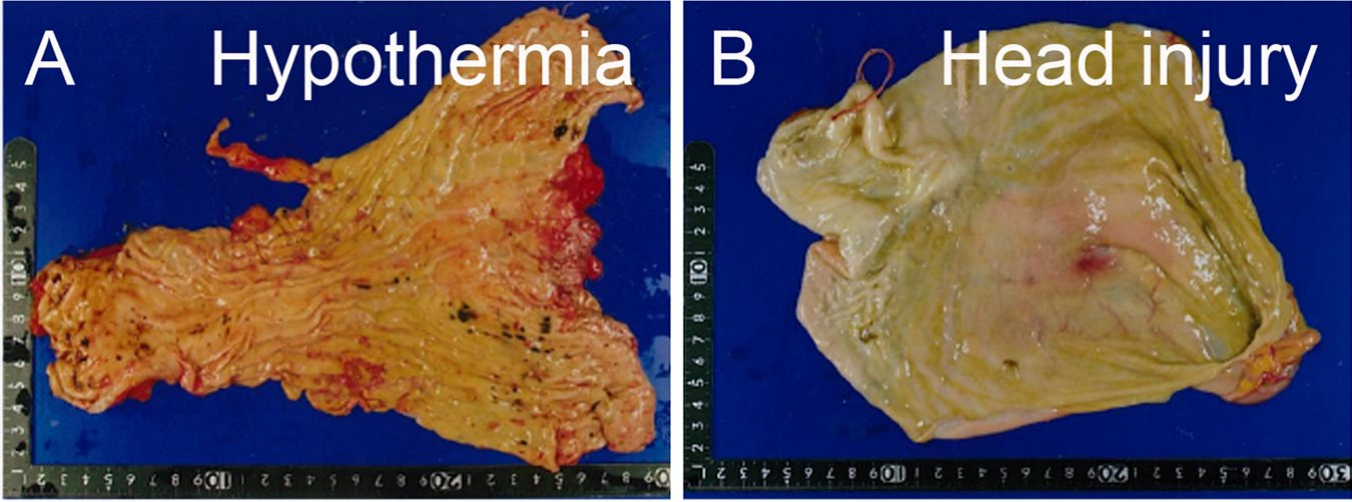
Wischnewski spots in fatal hypothermia. Representative pictures with or without Wischnewski spots in forensic autopsy cases performed at the Department of Legal Medicine, Osaka University. (**A**) shows Wischnewski spots observed in the stomach of a fatal hypothermia case. (**B**) shows a head injury case without the same findings.

Hypothermia is defined as a decrease in the body core temperature to below 35 °C and is associated with significant morbidity and mortality^1^. In the United States of America, about 1,500 people die annually from accidental hypothermia^2^. In Japan, there were 1,300–1,500 deaths from accidental hypothermia during 2017–2018 (ICD-10 code: X31) (vital statistics of Japan final data general mortality 2017, 2018). Central nervous system impairment and cardiac arrhythmias occur at core body temperatures below 33 °C^3,4^. Lethality has been reported to be 33% at core body temperatures of 20–30 °C and 59% at core body temperatures of under than 20 °C^5^. Despite severe physiological changes, pathological findings of fatal hypothermia at autopsy are slight and inconsistent. Importantly, gastric haemorrhage has never been recognized in accidental hypothermia in emergency medicine^1,6^. However, Wischnewski first reported in 1895 that the presence of numerous dark-brown coloured small spots similar to gastric haemorrhages, which are now termed “Wischnewski spots”, is frequently recognised in hypothermia-related death^7^ (Fig. 1). Since then, these spots have been considered to be a classical sign of fatal hypothermia, although their occurrence in fatal hypothermia has been described in the literature to vary between 40 and 100%^3,8–10^. Preuss *et al*. reported a case of fatal hypothermia with Wischnewski spots in ectopic gastric mucosa^11^. This case may show that the characteristics of the gastric mucosa are involved in the formation of Wischnewski spots, which cannot be confirmed in clinical cases with hypothermia^1,6^. In the present study, we hypothesised that fatal hypothermia alone may cause an imbalance between gastric acid secretion^12^ and the mucosal defence system^13^ on the gastric mucosa, leading to the formation of Wischnewski spots.

Gastrin, which is released from endocrine G cells, is a principal hormone regulating gastric acid secretion. Circulating gastrin stimulates both the synthesis and discharge of histamine in enterochromaffin-like (ECL) cells^14^. Gastrin and histamine bind to gastrin/cholecystokinin-B receptor (CCKBR) and histamine 2 receptor (HRH2), respectively, on the parietal cell surface and these parietal cells then undergo distinct dynamic morphological changes^14,15^. In resting parietal cells, tubulovesicles (TVs) exist in the intracellular compartments of the apical canalicular membrane and sequester H^+^,K^+^-ATPase pumps. After stimulation, the TVs move and bind to the canalicular membrane, exposing the H^+^,K^+^-ATPase pumps to the lumen and resulting in acid secretion^16,17^. Progastricsin (PGC), also known as pepsinogen C, is the precursor of pepsin C, which acts as a proteolytic enzyme in gastric secretions^18^. After its synthesis in the chief cells of the gastric mucosa, PGC is secreted into the gastric lumen where it is converted into pepsin C under acidic conditions^19^.

In the present study, we found that the cold loading of rat organotypic gastric mucosa slices lowered the pH of the culture media through the activation of gastric H^+^,K^+^-ATPase pumps and increased the secretion of pepsin C through inducing the expression of PGC.

## Results

### Cold stress induced gastric acidification and increased the expression of gastrin and histamine in cultured rat gastric slices

**Figure 2:**
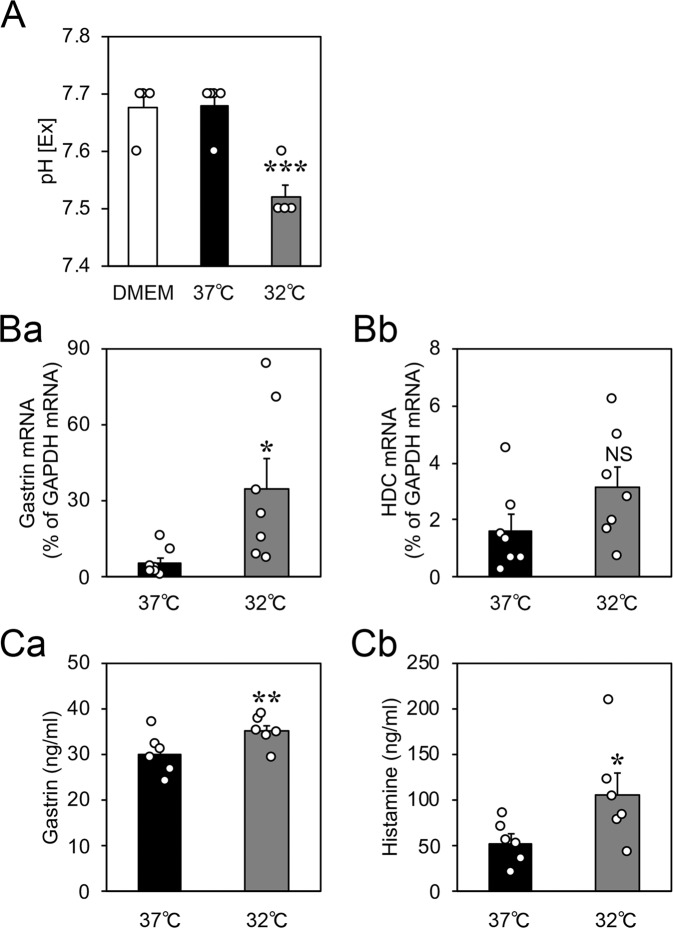
Effects of cold stress on the extracellular pH of the culture media and the expression of gastric acid secretagogues in cultured rat organotypic gastric slices. (**A**) The extracellular pH (pH [Ex]) in the culture medium of gastric slices decreased after incubation at 32 °C for 6 h (*n* = 5, rats). (**B**) The expression of gastrin mRNA increased after incubation at 32 °C for 3 h, but that of histidine decarboxylase mRNA did not change (*n* = 7, rats). (**C**) The abundances of gastrin protein and histamine in gastric slices were measured using enzyme-linked immunosorbent assay. The abundances of gastrin and histamine increased after incubation at 32 °C for 6 h (*n* = 6, rats). Data are expressed as the mean ± standard error of the mean (SEM). *, **, and *** indicate *p* < 0.05, 0.01, and 0.001, respectively, as compared to a control group incubated at 37 °C.

To assess the molecular mechanism of cold stress in the stomach, we isolated stomachs from rats and sliced them to 1–2 mm thickness. These slices were cultured at 37 °C (control) or 32 °C (cold) for 3–6 h. First, we assessed cold stress-induced gastric acid secretion by measuring the extracellular pH (pH [Ex]) in the culture medium. Gastric slices were cultured in the media with 25 mM 4-(2-hydroxyethyl)-1-piperazineethanesulfonic acid for 2.5 or 5.5 h to restrain a pH change, and then without it for a further 0.5 h. The pH [Ex] did not change after 3 h of incubation at 32 °C (Supplementary Fig. S1), but decreased after 6 h (Fig. 2A). Next, we investigated the expression of gastrin, which is one of the principal hormones regulating gastric acid secretion. The expression of gastrin mRNA increased after incubation at 32 °C for 3 h (Fig. 2Ba). The mRNA expression of histidine decarboxylase, which is the catabolic enzyme of histamine synthesis, was not changed by incubation at 32 °C (Fig. 2Bb). We also measured the abundances of gastrin and histamine in gastric slices using enzyme-linked immunosorbent assay (ELISA). Both gastrin and histamine significantly increased in abundance after incubation at 32 °C for 6 h (Fig. 2C). Therefore, a cold stress-induced increase in gastrin expression may upregulate the synthesis of histamine.

### Cold stress increased the expression of TV exocytosis-related factors and decreased the expression of cytokines in cultured rat gastric slices

**Figure 3:**
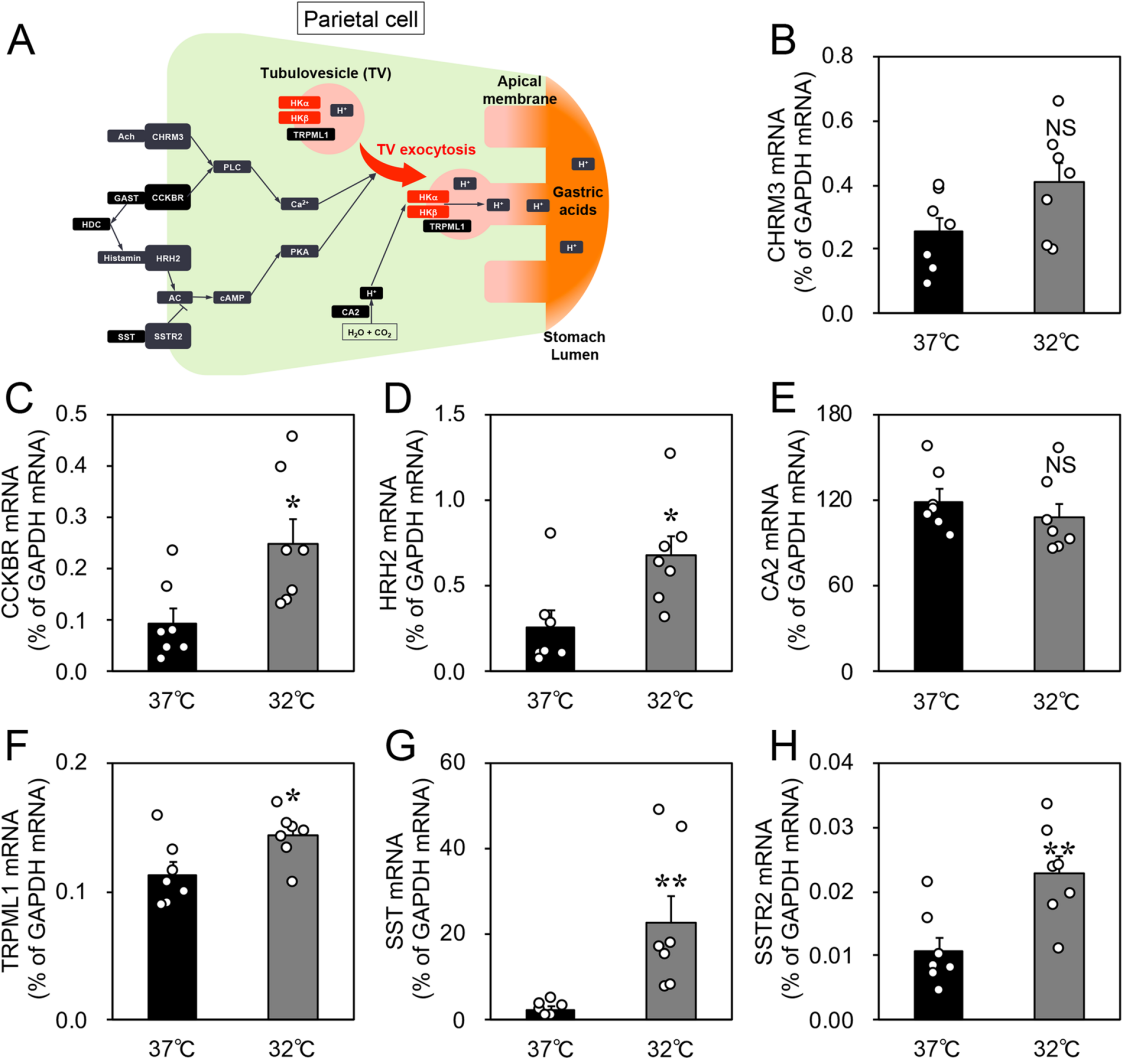
Effects of cold stress on the expression of tubulovesicle exocytosis-related factors on cultured rat organotypic gastric slices. (**A**) The exocytosis mechanism of tubulovesicles. Upon stimulation, tubulovesicles translocate and connect with the apical canalicular membrane, resulting in acid secretion. (**B**–**H**) Incubation at 32 °C for 3 h increased the expression of mRNAs encoding the cholecystokinin B receptor (CCKBR), histamine receptor (HRH2), transient receptor potential mucolipin-1 (TRPML1), somatostatin (SST), and somatostatin receptor 2 (SSTR2). The expression of mRNAs encoding the acetylcholine receptor (CHRM3) and carbonic anhydrase (CA2) did not change. Data are expressed as mean ± SEM (*n* = 7, rats). * and ** indicate *p* < 0.05 and 0.01, respectively, as compared to a control group incubated at 37 °C. NS, not significantly different.

**Figure 4:**
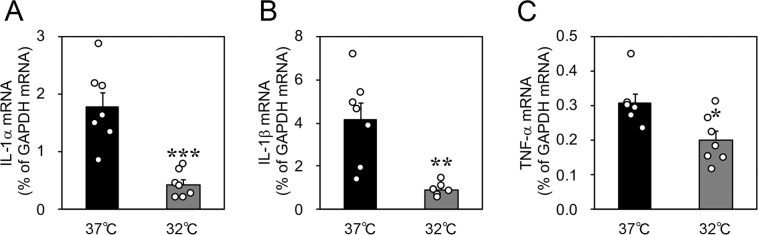
Effects of cold stress on the expression of cytokines in cultured rat organotypic gastric slices. The expression of antisecretory genes encoding interleukin-1α (IL-1α), interleukin-1β (IL-1β), and tumor necrosis factor-α (TNF-α) decreased after incubation at 32 °C for 3 h. Data are expressed as the mean ± SEM (*n* = 7, rats). *, **, and *** indicate *p* < 0.05, 0.01, and 0.001, respectively, as compared to a control group incubated at 37 °C.

Figure 3A shows the expression of tubulovesicle (TV) exocytosis-related factors in parietal cells. The expression of prosecretory genes encoding the acetylcholine receptor CHRM3, the cholecystokinin B receptor CCKBR, the histamine receptor HRH2, the carbonic anhydrase CA2, and the transient receptor potential mucolipin-1 TRPML1 was determined by quantitative polymerase chain reaction (qPCR). Furthermore, we also determined the expression of antisecretory genes encoding somatostatin (SST) and SST receptor 2 (SSTR2). The expression of the three prosecretory genes encoding CCKBR, HRH2, and TRPML1 (Fig. 3C,D,F) and the two antisecretory genes encoding SST and SSTR2 increased after incubation at 32 °C for 3 h (Fig. 3G,H). SST is produced by D cells but not parietal cells. Interleukin-1α (IL-1α), interleukin-1β (IL-1β), and tumour necrosis factor-α (TNF-α) have been reported to inhibit the secretion of gastric acid^20–23^ and thus we assessed the effects of cold stress on the expression of these cytokines. The expression of these antisecretory cytokines significantly decreased after incubation at 32 °C for 3 h (Fig. 4). These data may show that a cold stress-induced increase in the expression of prosecretory factors and decrease in the expression of antisecretory cytokines facilitated the increased abundance of H^+^,K^+^-ATPase protein and the apical canalicular membrane fusion of TVs.

### Cold stress increased the expression of H^+^,K^+^-ATPase in cultured rat gastric slices

**Figure 5:**
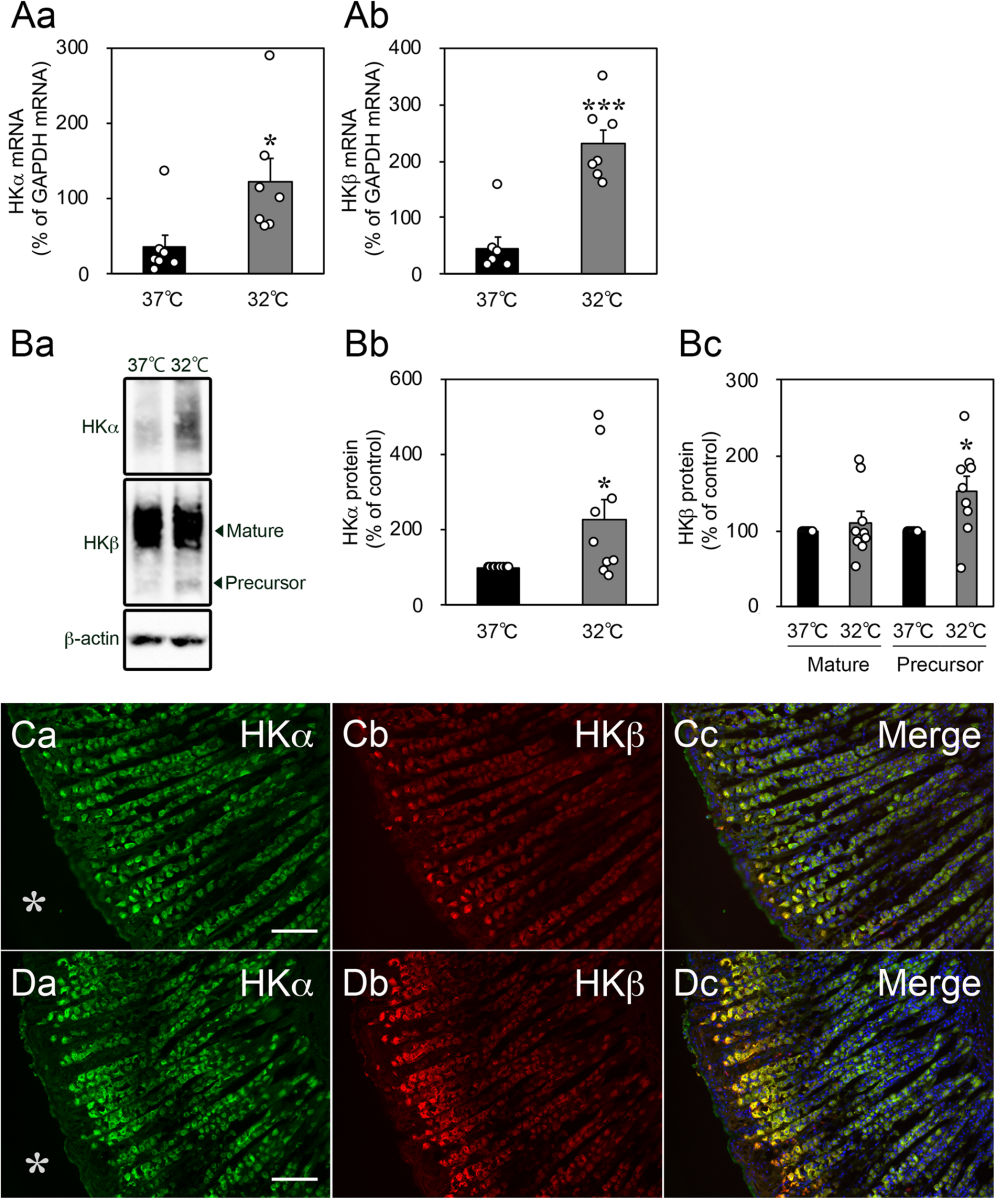
Effect of cold stress on gastric H^+^,K^+^-ATPase expression in cultured rat organotypic gastric slices. (**A**) Incubation at 32 °C for 3 h increased the mRNA expression of the H^+^,K^+^-ATPase α-subunit (HKα) and β-subunit (HKβ). Data are expressed as the mean ± SEM (*n* = 7, rats). * and *** indicate *p* < 0.05 and 0.001, respectively, as compared to a control group incubated at 37 °C. (**B**) The expression levels of total HKα and HKβ-precursor protein increased after incubation at 32 °C for 6 h. Data are expressed as the mean ± SEM (*n* = 9, rats). *p < 0.05 as compared to a control group incubated at 37 °C. (**C**,**D**) The abundances of the HKα (green) and HKβ (red) proteins increased after incubation at 32 °C for 6 h, especially in the luminal parietal cells (C, 37 °C and D, 32 °C). Hoechst 33258 staining (blue) revealed nuclei in the merged micrographs. Asterisks indicate the lumen of the stomach. Scale bar, 100 µm.

Next, we investigated the expression of gastric H^+^,K^+^-ATPase, which mediates the secretion of gastric acid into the lumen. The mRNA expression of HKα and HKβ significantly increased after incubation at 32 °C for 3 h (Fig. 5A), and the abundance of the HKα and HKβ proteins also increased after incubation at 32 °C for 6 h (Fig. 5B and Supplementary Fig. S2). In the gastric mucosa, the H^+^,K^+^-ATPase pumps are expressed specifically in parietal cells and are therefore used as markers for this cell type. Immunohistochemistry showed that the expression of these proteins increased in the parietal cells on the luminal side (Fig. 5C,D).

### Cold stress induced morphological changes of parietal cells in cultured gastric slices

**Figure 6:**
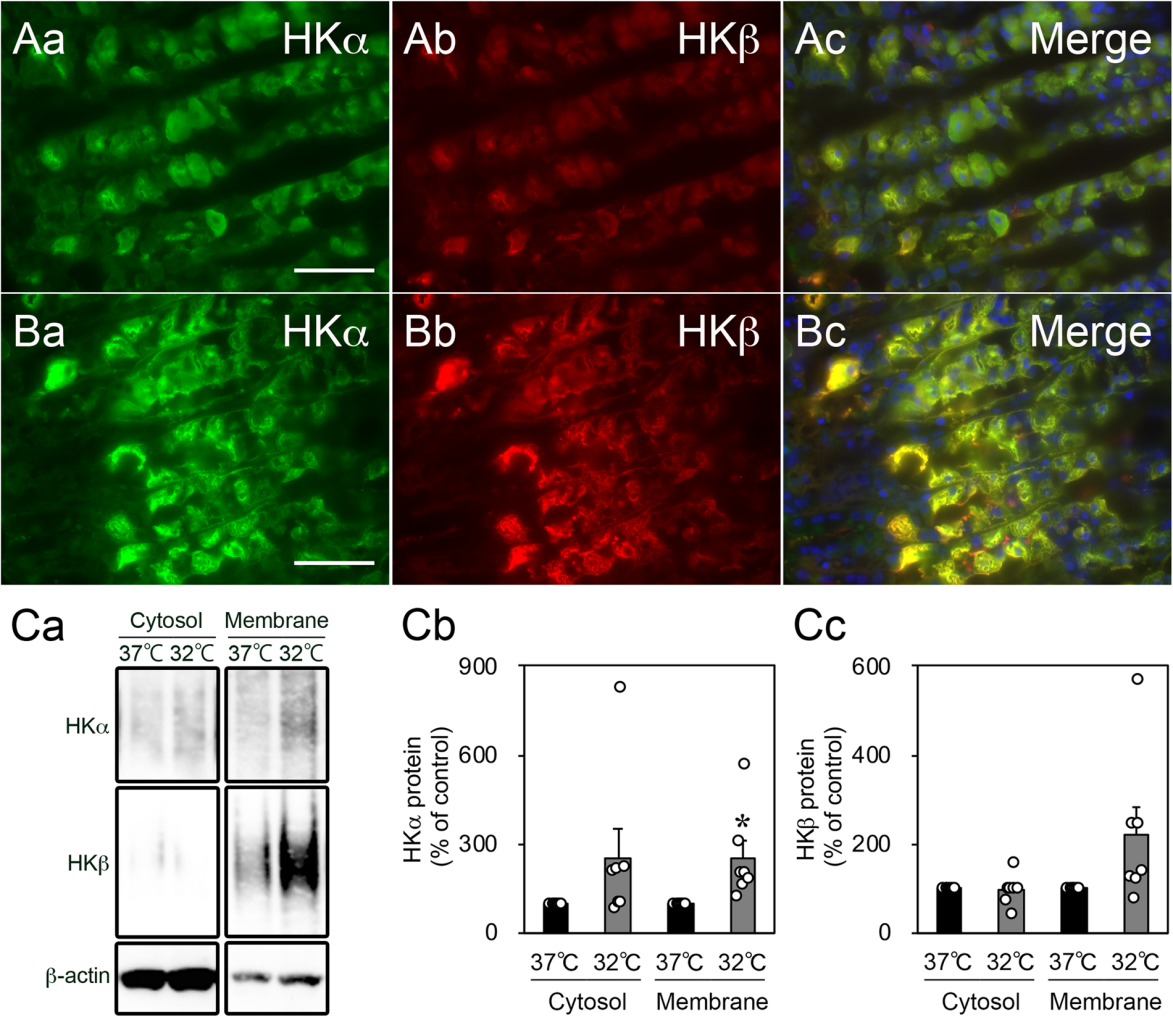
Effects of cold stress on the morphology of parietal cells in cultured rat organotypic gastric slices. (**A**,**B**) The morphology of parietal cells changed from the resting state to the stimulated state after incubation at 32 °C for 6 h (A, 37 °C and B, 32 °C). Upon incubation at 32 °C, the tubulovesicles formed a reticulated meshwork. The green and red colours show HKα and HKβ, respectively. Hoechst 33258 staining (blue) revealed nuclei in the merged micrographs. Scale bar, 50 µm. (**C**) The expression levels of the HKα and HKβ proteins in the cytosol and membrane. HKα expression in the membrane significantly increased after incubation at 32 °C for 6 h (Cb). Data are expressed as the mean ± SEM (*n* = 7, rats). *p < 0.05 as compared to a control group incubated at 37 °C.

Under stimulation with secretagogues (gastrin, histamine, and acetylcholine), parietal cells undergo striking morphological changes and TVs translocate to fuse with the apical canalicular membrane (Fig. 3A). Therefore, we assessed the effects of cold stress on the morphology of these cell types in gastric slices. After incubation at 32 °C (cold stress) for 6 h, the morphology of the parietal cells expressing the HKα and HKβ proteins changed in comparison with the control (Fig. 6A,B). There was no histological change under hematoxylin and eosin (HE) staining between 32 °C and 37 °C group (Supplementary Fig. S3). Next, to investigate whether TVs fuse to the apical membrane after incubation at 32 °C, we extracted the cytosolic and cell membrane fractions of gastric slices. The abundance of the HKα-membrane (HKα expressed in membrane fraction) protein significantly increased after incubation at 32 °C for 6 h (Fig. 6Ca,b, and Supplementary Fig. S4). The abundances of the HKα-cytosol and HKβ-membrane proteins also increased, but did not reach statistical significance. (Fig. 6Cc).

### Cold stress increased the expression of PGC and pepsin C in cultured gastric slices

**Figure 7:**
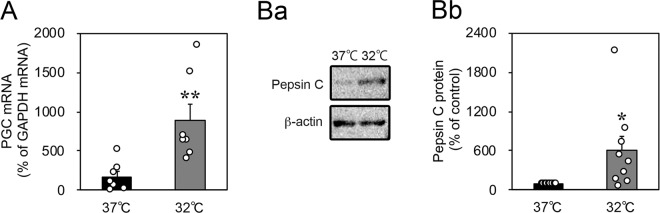
Effects of cold stress on the expression of progastricsin and pepsin C in cultured rat organotypic gastric slices. (**A**) The mRNA expression of progastricsin significantly increased after incubation at 32 °C for 3 h (*n* = 7, rats). (**B**) The abundance of pepsin C protein increased after incubation at 32 °C for 6 h (*n* = 9, rats). Data are expressed as the mean ± SEM. * and ** indicate *p* < 0.05 and 0.01, respectively, as compared to a control group incubated at 37 °C.

Next, we investigated the expression of proteolytic enzymes in cultured gastric slices. The mRNA expression of PGC, which is a precursor of pepsin C, increased after incubation at 32 °C for 3 h (Fig. 7A). Furthermore, incubation at 32 °C for 6 h also facilitated the conversion of PGC into pepsin C protein (Fig. 7B and Supplementary Fig. S5). These data show that cold stress increased the production of proteolytic enzymes in the gastric lumen.

## Discussion

The organotypic slice culture method has been widely used in studies on the brain and heart^24,25^. In the present study, we used an organotypic stomach slice culture to clarify the mechanisms underlying the development of Wischnewski spots on the gastric mucosa, which is often observed in fatal hypothermia. This culture method allows the micro environment and structure of gastric wall to exert its potential influence on gastric secretion, and we could perform examinations with the experimental and control treatments using paired tissues from the same individual rat. Moreover, this culture method has the critical advantage that it is free from systemic influences such as blood pressure, blood flow, and central nervous control that can complicate the performance and interpretation of *in vivo* experiments. White blood cell (WBC) infiltration, which can be observed in histology of inflammation diseases, was not supposed to appear in the slice without blood. However, WBCs were still observed histopathology (Supplementary Fig. S3Aa,Ba), suggesting that residual WBCs cannot be removed.

Gastrin, which is released by endocrine G cells, is the principal hormone regulating gastric acid secretion. Gastrin stimulates parietal cells both directly and indirectly as well as both the synthesis and secretion of histamine in enterochromaffin-like (ECL) cells^14^. In the stomach, the gastrin receptor CCKBR is expressed on both parietal and ECL cells^14,15^. ECL cells regulate acid secretion in parietal cells by releasing histamine, binding to H2 receptors on parietal cells. Thus, gastrin induces acid secretion directly by binding to CCKBR on parietal cells and indirectly by stimulating histamine release from ECL cells^26^. Furthermore, it has been reported that gastrin increased the mRNA expression of HKα and HKβ as detected through the transcriptional profiling of gastrin-regulated genes in gastrin-deficient mice^27,28^. Our data also showed that the expression of gastrin, histamine, HKα, and HKβ was increased by cold stress (Figs. 2B,C and 6). Therefore, acidification and expression of H^+^,K^+^-ATPase may occur as a result of the cold stress-induced increase of gastrin expression and the gastrin-induced increase of histamine expression.

Furthermore, upon stimulation with secretagogues (gastrin, histamine, and acetylcholine), parietal cells undergo dramatic morphological changes through the exocytosis mechanism of TVs (Fig. 3A)^12,17,29^. As shown in Fig. 6, cold stress induced dynamic morphological changes in parietal cells and led to the fusion of TVs on the canalicular membrane. We thought that the cold stress-induced increase in gastrin expression might result in acidification through inducing the activation of parietal cells and increasing the expression of H^+^,K^+^-ATPase. The expression of SST and its receptor (SSTR2), which are antisecretory factors, also increased under cold stress (Fig. 3G,H). SST was previously reported to inhibit acid secretion by about 30 to 40%^30^. Therefore, the increased expression of these antisecretory factors might not substantially inhibit cold stress-induced acidification.

The cytokines IL-1α, IL-1β, and TNF-α inhibit gastric acid secretion^20–23^. In a previous study, neither IL-1β nor TNF-α had any effect on the ligand binding activities of HRH2 or CCKBR^21^. The inhibitory actions of these cytokines are not thought to be secretagogue-specific, although they characteristically inhibit gastric acid secretion induced by activation of the phospholipase C/Ca^2+^/inositol phosphate pathway (e.g., gastrin and carbachol) more strongly than that induced by histamine^21,23^. Figure 4 shows that the expression of these cytokines significantly decreased under cold stress, suggesting that the downregulation of these cytokines may also contribute to gastric acidification in hypothermic conditions.

Furthermore, cold stress promoted the production of proteolytic enzymes together with the induction of acidification (Fig. 7). PGC was synthesised in the chief cells, secreted into the gastric lumen, and then converted into pepsin C under acidic conditions^19^. Our results confirmed that the excessive production of pepsin C was caused by a synergistic effect of the cold stress-induced acidification and the increase in the expression of PGC. Wischnewski spots could not be taken from the stomach surface. Therefore, these findings suggested that Wischnewski spots were formed by submucosal haemorrhages from microvessels that were damaged by pepsin. Through an immunohistochemical examination, Tsokos *et al*. found that Wischnewski spots represented haemoglobin released from destroyed erythrocytes^9^. Their findings may support our results.

The incidence of expression of Wischnewski spots have been 40 to 100% in fatal hypothermia cases^3,8–10^. The variation of its occurrence among cases of fatal hypothermia may rely upon the consideration of factors such as the circumstances of the death and the findings at the scene of death. In addition, differences may result from severe atrophic gastritis or *Helicobacter pylori* infection, which may cause a low secretion of gastric acid^31^. Wischnewski spots have been recognized in cases of non-hypothermic diabetic ketoacidosis^32^ and fatal burn^33^. Nevertheless, the genesis and pathophysiology of these spots in cases of fatal hypothermia remain unclear. Furthermore, the levels of cold stress-induced acidification and proteolytic enzyme expression may also influence the occurrence of Wischnewski spots.

**Figure 8:**
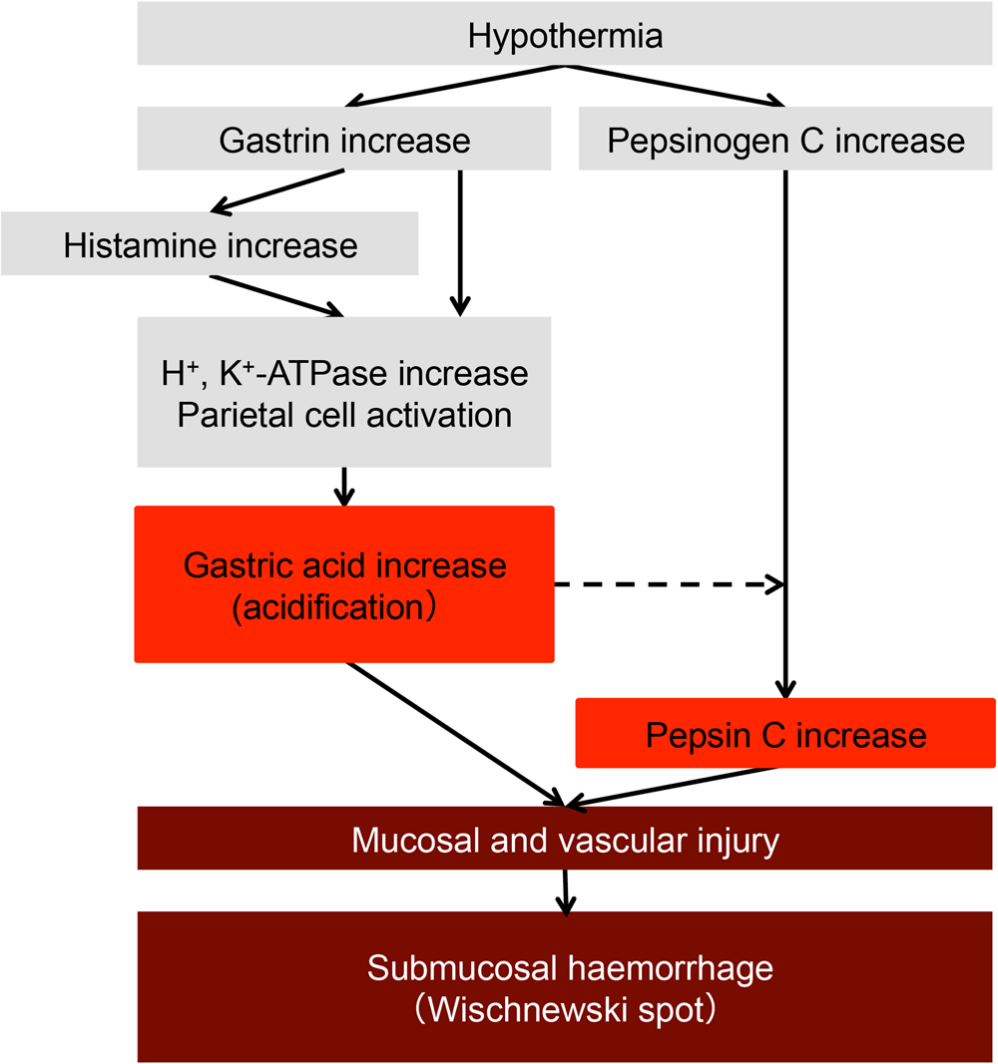
Formation mechanism of Wischnewski spots elucidated by the present study. Hypothermia increases the secretion of gastric acid and pepsin C, which might subsequently damage the gastric mucosa and microvessels. These events may induce the formation of Wischnewski spots.

In conclusion, the present study clarified that cold stress promoted the secretion of gastrin from G cells and the synthesis of histamine by ECL cells (Fig. 8). The cold stress-induced release of gastrin and histamine increased the expression of ATPases in the apical canalicular membrane, resulting in acid secretion. Cold stress also promoted the secretion of PGC from the chief cells and increased the expression of pepsin C in the gastric lumen. These phenomena induced by cold stress may contribute to the formation of Wischnewski spots.

## Materials and Methods

### Animals

All animal experiments were approved by the Animal Ethics Committee of Osaka University Graduate School of Medicine, and were performed according to the guideline outlined in the Guide for the Institute of Experimental Animal Sciences Faculty of Medicine, Osaka University. Eight-week-old male Wistar rats (Kiwa Laboratory Animals, Wakayama, Japan) were used for the slice culture experiment. Rats were fasted 6 h before the experiment with free access to tap water.

### Gastric slice culture

After the rats were killed using CO_2_ gas, the stomach was isolated and washed with warm phosphate-buffered saline (PBS) at 37 °C. Slices of 1–2 mm thickness were cut longitudinally from the area between the cardia and pylorus using Alto™ brain matrices (CellPoint Scientific, Gaithersburg, MD, USA). The slices were then cultured on a 100-µm pore mesh in culture medium comprised of low-glucose Dulbecco’s modified Eagle’s medium (Wako Pure Chemical Industries, Osaka, Japan) supplemented with 10% fetal bovine serum (Gibco, Grand Island, NY, USA), 1% Penicillin-Streptomycin-Amphotericin B suspension (Wako Pure Chemical Industries). 25 mM 4-(2-hydroxyethyl)-1-piperazineethanesulfonic acid was added to prevent pH change in the culture medium. The culture environment was maintained using a CO_2_ incubator (Panasonic, Osaka, Japan) with 5% CO_2_ and the initial setting of 37 °C as “control” or 32 °C as “cold stress”. Gastric slices were cultured in the media with 25 mM 4-(2-hydroxyethyl)-1-piperazineethanesulfonic acid. The medium was replaced every 1 h to avoid pH change except for the experiments of pH [Ex] and ELISA. The pH [Ex] in the culture medium was measured at 3 or 6 hours using compact pH meter LAQUAtwin (Horiba, Kyoto, Japan).

### Quantitative real-time RT-PCR (qRT-PCR)

Total RNA in the slices was isolated using ISOGEN^®^ reagent (Nippon Gene, Tokyo, Japan). Gastric slices were homogenized using a bead homogenizer (Taitec, Saitama, Japan) with ISOGEN^®^ reagent. The RNA was reverse-transcribed using ReverTra Ace^®^ qPCR RT master mix with gDNA remover (Toyobo, Osaka, Japan). qPCR analysis was performed in duplicate using a CFX Connect™ real-time system (Bio-Rad Laboratories, Hercules, CA, USA) along with SYBR^®^ premix Ex Taq™ II (Tli RNaseH Plus) (Takara Bio, Kusatsu, Japan). All gene-specific mRNA expression values were normalized to the expression value of glyceraldehyde-3-phosphate dehydrogenase (GAPDH) mRNA. The primer sequences for each gene are listed in Supplementary Table S1.

### Determination of gastrin and histamine levels

Gastric slices were cultured at 37 °C or 32 °C for 6 h and then homogenized using a bead homogenizer with 0.5 mL PBS. The suspensions were centrifuged at 15,000 rpm for 20 min at 4 °C. Gastrin and histamine levels in the supernatant were determined using a rat gastrin 1 ELISA kit (Abcam, Cambridge, UK) and a histamine ELISA kit (Abcam), respectively, according to each manufacturer’s instructions.

### Protein extraction

To analyse total protein expression, the slices were homogenized with RIPA buffer (Wako Pure Chemical Industries). The suspension was centrifuged at 15,000 rpm for 20 min at 4 °C and the supernatant was collected. To separate cytosol and membrane proteins, the slices were homogenized in a solution containing 250 mM sucrose, 1 mM egtazic acid, and 5 mM Tris-HCl (pH 7.4)^17^. The suspension was centrifuged at 1,000 × *g* for 10 min at 4 °C, and the supernatant was further centrifuged at 13,500 × *g* for 30 min at 4 °C. The supernatant and pellet were used as cytosol and membrane fractions, respectively. The concentration of protein in each sample was determined using a bicinchoninic acid protein assay kit (Takara Bio), and 3 μg of each protein sample was mixed with Laemmli buffer and analysed by western blotting.

### Immunoblotting

The lysates were separated by sodium dodecyl sulphate polyacrylamide gel electrophoresis using a 10% acrylamide gel, transferred to nitrocellulose membranes, and immunoblotted with antibodies against β-actin (1:1000; mouse monoclonal, clone AC-15; Sigma-Aldrich, St. Louis, MO, USA), H^+^,K^+^-ATPase α-subunit (HKα; 1:500; mouse monoclonal; MBL, Nagoya, Japan), H^+^,K^+^-ATPase β-subunit (HKβ; 1:500; mouse monoclonal; Sigma-Aldrich), and pepsin C (1:500; mouse monoclonal, E-9; Santa Cruz Biotechnology, CA, USA). To enhance the signal, Can Get Signal^®^ solution (Toyobo, Osaka, Japan) was used. After incubation with a horseradish peroxidase-linked secondary antibody (1:5000; GE Healthcare, Marlborough, MA, USA), immunoreactions were visualised using Western BLoT Chemiluminescence HRP Substrate (Takara Bio). Immunoreactive bands were analysed by densitometry using Adobe Photoshop (Adobe Systems Incorporated, San Jose, CA, USA). The densitometry data were standardised against the immunoreactivity of β-actin.

### Immunohistochemistry

Gastric tissues were fixed using 4% paraformaldehyde in phosphate buffer solution (Wako Pure Chemical Industries) at 4 °C overnight. Next, these tissues were immersed in 15% sucrose in PBS at 4 °C overnight, frozen in dry ice powder, and sliced into 10-μm-thick sections. Sections were incubated with antibodies against HKα (1:100; rabbit polyclonal; Calbiochem, La Jolla, CA, USA) and HKβ (1:100; mouse monoclonal; Sigma-Aldrich), and then subsequently incubated with DyLight^®^ 488 and DyLight^®^ 649-labelled secondary antibodies (Invitrogen, Carlsbad, CA, USA). Hoechst 33258 (Invitrogen) was used for nuclear staining. The immunostained specimens were observed with a fluorescence microscope (BZ-X710; Keyence, Osaka, Japan).

### Hematoxylin and eosin staining

Gastric tissues were fixed using 4% paraformaldehyde in phosphate buffer solution (Wako Pure Chemical Industries) at 4 °C overnight. Next, these tissues were immersed in 15% sucrose in PBS at 4 °C overnight, frozen in dry ice powder, and sliced into 10-μm-thick sections. Sections got fully dipped with Mayer’s hematoxylin (Wako Pure Chemical Industries) and eosin Y (Wako Pure Chemical industries), sequentially. The HE stained specimens were observed with a light field microscopy (BZ-X710; Keyence).

### Statistical analysis

Values are expressed as the mean ± standard error of the mean. Data were analysed using two-tailed Student’s *t*-test (unpaired or paired) or analysis of variance with Tukey’s post-hoc test among groups. The threshold for significance was set at *p* < 0.05.

## Supplementary information

Supplementary information
