# Supplementary material for: Molecular mechanisms of Wischnewski spot development on gastric mucosa in fatal hypothermia: an experimental study in rats
Source: Sci Rep. 2020 Feb 5;10:1877. doi: 10.1038/s41598-020-58894-8 (PMC7002760; doi:10.1038/s41598-020-58894-8)
Supplement: Supplementary file 1 — Supplementary information [file 41598_2020_58894_MOESM1_ESM.pdf]

Molecular mechanisms of Wischnewski spot development on gastric mucosa in fatal hypothermia: an experimental study in rats

Chihpin Yang[1], Kana Sugimoto[1]*, Yukie Murata[1], Yuichiro Hirata[1], Yu Kamakura[1], Yoshihisa Koyama[2], Yohei Miyashita[1], Kentaro Nakama[1], Kazuma Higashisaka[1], Kazuo Harada[1], Ryuichi Katada[1], Hiroshi Matsumoto[1]*.

Affiliations:

[1] Department of Legal Medicine, Osaka University Graduate School of Medicine.

[2] Department of Neuroscience and Cell Biology, Osaka University Graduate School of Medicine.

*Corresponding authors:

Kana Sugimoto, Department of Legal Medicine, Osaka University Graduate School of Medicine. 2-2 Yamadaoka, Suita, Osaka 565-0871, Japan.

E-mail: sugikana@legal.med.osaka-u.ac.jp

Hiroshi Matsumoto, Department of Legal Medicine, Osaka University Graduate School of Medicine. 2-2 Yamadaoka, Suita, Osaka 565-0871, Japan.

E-mail: matsumo@legal.med.osaka-u.ac.jp

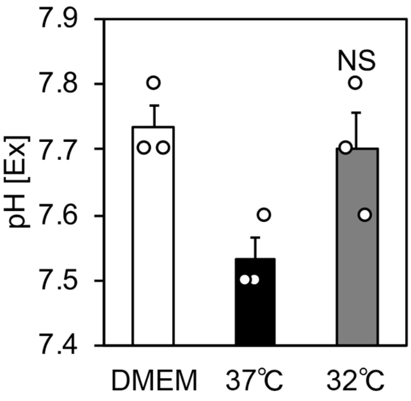

Supplementary Fig. S1. Effects of cold stress on the extracellular pH of the culture media.

The extracellular pH (pH [Ex]) in the culture medium of gastric slices decreased after incubation at 32 °C for 3 h (n = 3 rats). The pH [Ex] was not changed by incubation at 32 °C. NS, not significantly different.

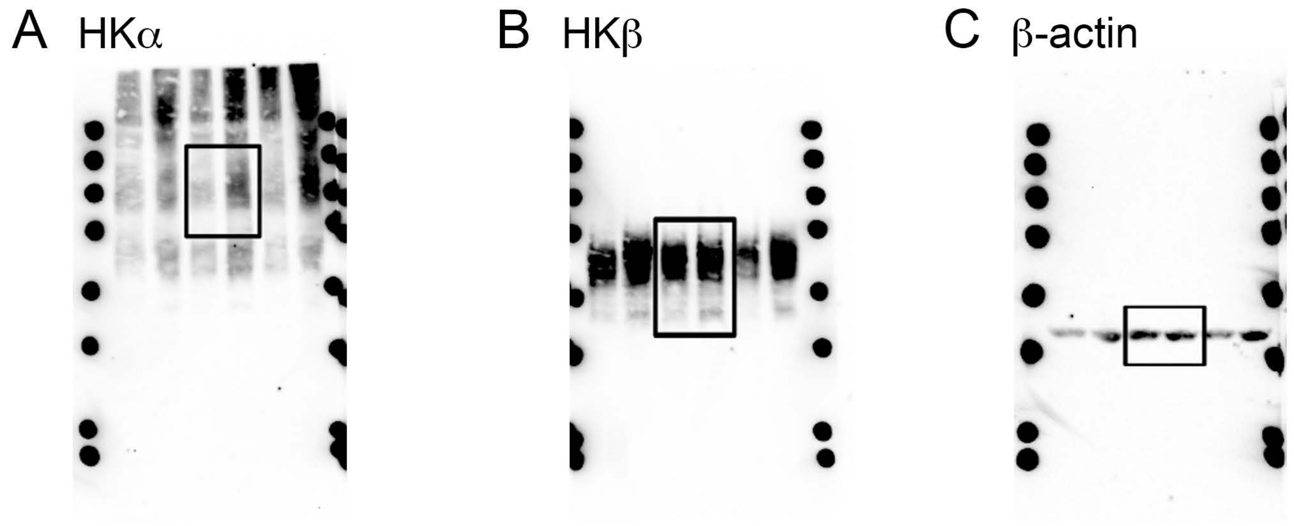

Supplementary Fig. S2. Western blot image of Fig. 5Ba-proteins.

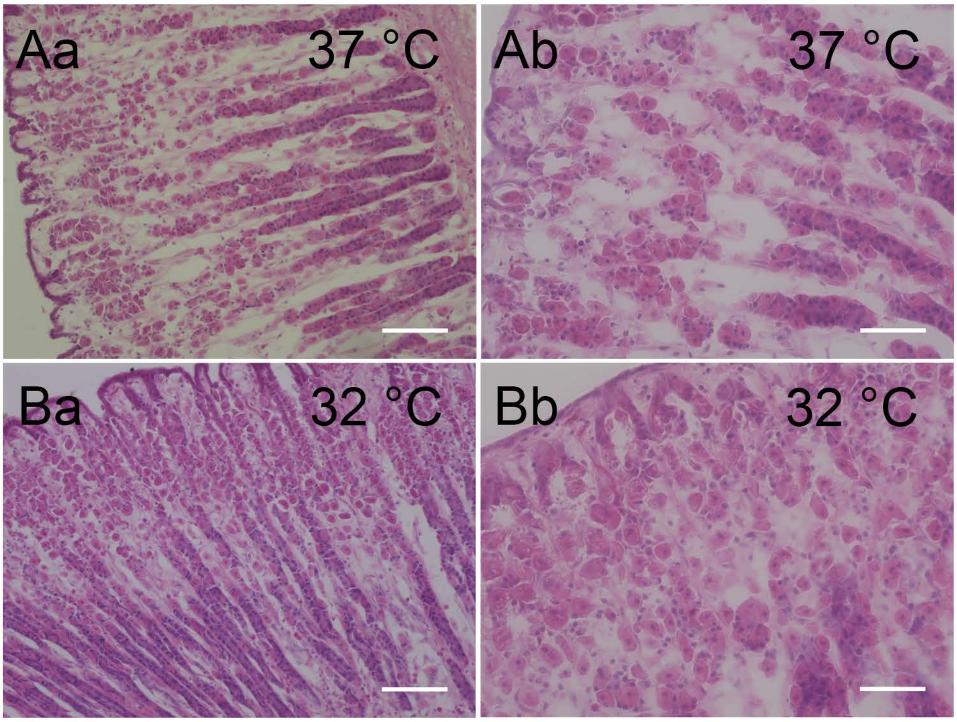

Supplementary Fig. S3. Effects of cold stress on the morphology of gastric cells in cultured rat organotypic gastric slices.

Scale bar, 100 μm (Aa and Ba) and 50 μm (Ab and Bb).

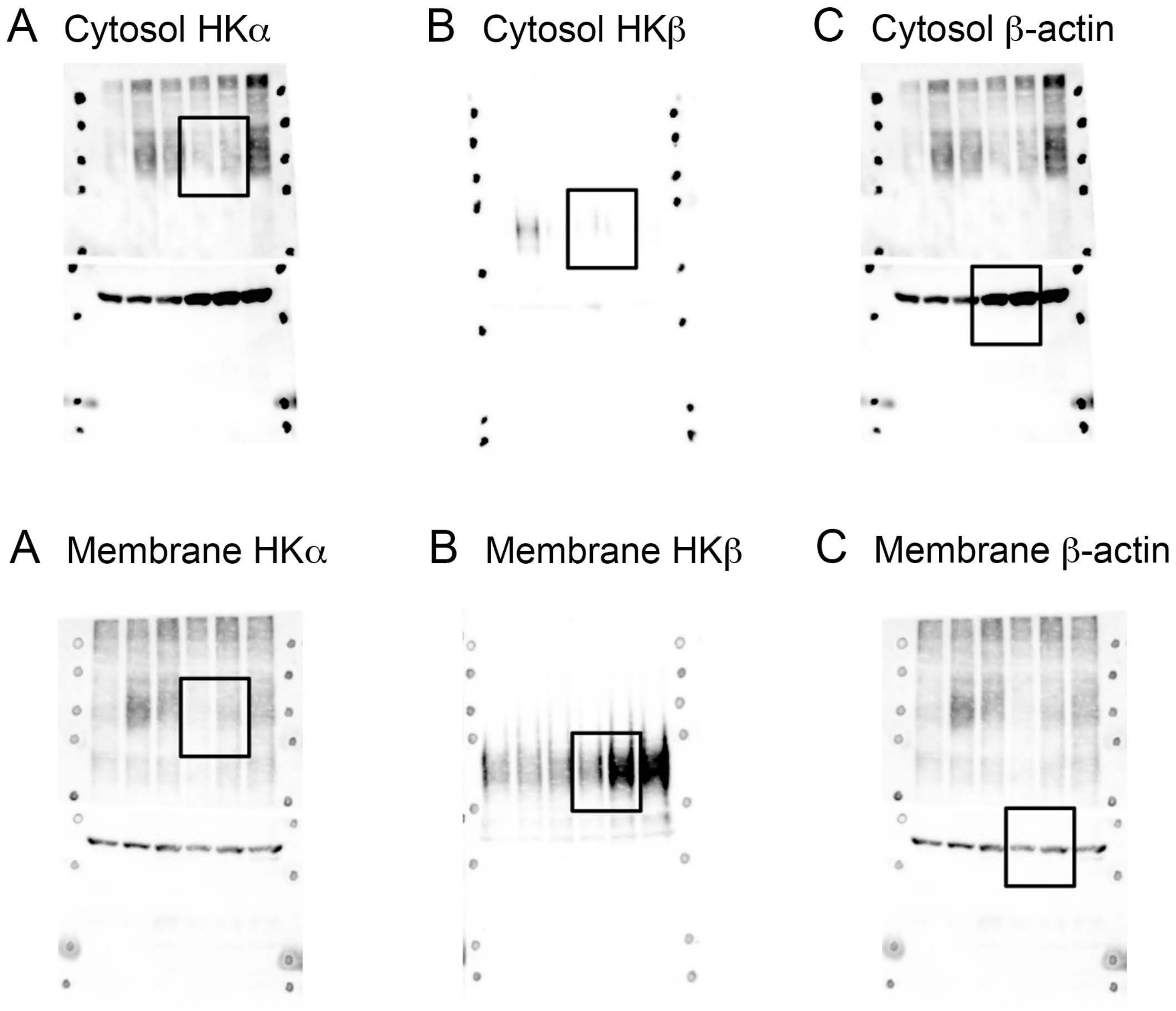

Supplementary Fig. S4. Western blot image of Fig. 6Ca-proteins.

A Pepsin C

B β-actin

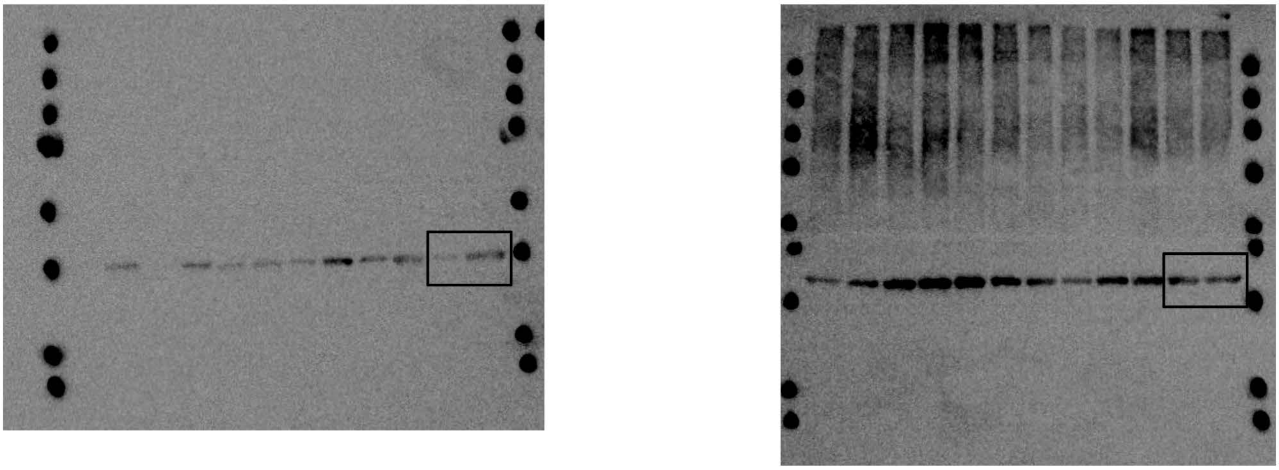

Supplementary Fig. S5. Western blot image of Fig. 7Ba-proteins.

Gene	Sense	Anti-sense
CA2	ACTCCCAGGACTTTGCAGTG	CCCCATATTTGGTGTTCCAG
CCKBR	GAAACGTGCTCATCATCGTG	TGAGGTAGGAAATGGCCTTG
CHRM3	TGCTAGCCTTCATCATCACG	TCACACTGGCACAAGAGGAG
GAST	ATGCCTCGACTGTGTGTGTG	CATTGGTGGCCTCTGTTTCT
HDC	ATGTGAAGCCTGGGTACCTG	GTGAACCCCAAGCAGTTGAT
HKα	GCACCCCTGAGTACGTGAAG	ACCACAACCACAGCAATGAG
HKβ	ATCCCTACACCCCCGACTAC	GGTATGGTTCGGAGCAGAAA
HRH2	AACAGCAGAAATGGGACCAG	TGGCTTTGTGCTCTCTGATG
IL-1α	TCGGGAGGAGACGACTCTAA	GAAAGCTGCGGATGTGAAGT
IL-1β	CTGTGACTCGTGGGATGATG	AGGGATTTTGTCGTTGCTTG
PGC	ACACTCTGACCGTCCAAAGC	CTGGCTGCCAAGGTAGACTC
SST	ATGCTGTCCTGCCGTCTC	AGGGCATCGTTCTCTGTCTG
SSTR2	GCTACGCCAAGATGAAAACC	TCATGACCGTCAAGCAGAAG
TNF-α	CCCAGACCCTCACACTCAGAT	TTGTCCCTTGAAGAGAACCTG
TRPML1	AAACACCCCAGTGTCTCCAG	ACCAGCCATTGACAAACTCC

Supplementary Table S1. Oligonucleotide primers for real-time RT-PCR
